# Influence of management on susceptibility of Nordic boreal forests to damage by natural disturbances

**DOI:** 10.1007/s13280-026-02394-y

**Published:** 2026-05-19

**Authors:** Teresa Fustel, Karin Öhman, Maartje Klapwijk, Tomas Lämås, Niklas Björklund, Johanna Boberg, Michelle Cleary, Annika Felton, Adam Felton, Stephanie Jonsson, Wiebke Neumann, Carin Nilsson, Åke Olson, Jonas Rönnberg, Jeannette Eggers

**Affiliations:** 1 Department of Forest Resource Management, Swedish University of Agricultural Sciences, 901 83 Umeå, Sweden; 2 Department of Ecology, Swedish University of Agricultural Sciences, 750 07 Uppsala, Sweden; 3 Department of Forest Mycology and Plant Pathology, Swedish University of Agricultural Sciences, 750 07 Uppsala, Sweden; 4 Southern Swedish Forest Research Centre, Swedish University of Agricultural Sciences, 234 22 Lomma, Sweden; 5 Department of Electrical Engineering, Mathematics, and Science, Gävle University College, 80176 Gävle, Sweden; 6 Department of Wildlife, Fish, and Environmental Studies, Swedish University of Agricultural Sciences, 901 83 Umeå, Sweden; 7 Skogsstyrelsen (Swedish Forest Agency), Box 63, 28121 Hässleholm, Sweden; 8 Skogforsk (The Forestry Research Institute of Sweden), Uppsala Science Park, 751 83 Uppsala, Sweden

**Keywords:** Boreal forest, Forest management adaptation, Forest susceptibility, Mitigation, Natural disturbance, Production forests

## Abstract

Natural disturbances are fundamental in shaping forest ecosystems, yet management decisions in production forests can aggravate or mitigate their impacts. Understanding the interplay between forest adaptation strategies and susceptibility to damage is important for sustainable forest management. Here, we review the scientific literature to explore the relationship between natural disturbances and the impact of forest adaptation strategies on the forest susceptibility to damage. To do so we reviewed seven different adaptation strategies (mixed-species stands, shorter rotations, longer rotations, few or no thinnings, logging residue removal, prescribed burning and uneven-aged forestry), and how they affect forest susceptibility to damage by ten different disturbances. Each of the strategies offers opportunities to improve the forest's resilience to damage in different ways and against specific disturbances. A combination of these strategies may have a greater impact on reducing forest susceptibility to damage. The review informs the development of adaptation strategies considering relevant forest characteristics coupled to natural disturbances.

**Supplementary Information:**

The online version contains supplementary material available at https://doi.org/10.1007/s13280-026-02394-y.

## Introduction

Natural disturbances play a fundamental role in forest ecosystem dynamics, changing the availability of resources and generating diverse forest characteristics (Attiwill 1994; Svoboda et al. 2014). However, disturbance dynamics in Europe’s forests have intensified in recent years, producing substantial economic losses in forests managed for timber production (e.g. Senf and Seidl 2021; Aakala et al. 2023; Patacca et al. 2023). During the first decade of the twenty-first century, damage in European forests was substantially higher for a number of disturbances, such as windstorms and spruce bark beetle (*Ips typographus* L. (*Coleoptera: Curculionidae, Scolytinae*)) outbreaks, compared to the end of the twentieth century (Seidl et al. 2014). This increase in frequency and intensity of natural disturbances in forest ecosystems has been partially attributed to climate change (e.g. Lemke et al. 2007; Lindner et al. 2010). For example, an increase in the overall average temperature on land surface has been linked to more severe extreme weather events (IPCC 2023), such as droughts, which in turn decrease the trees' vigour and increase the chances of insect pest attacks like spruce bark beetle, leading to potential outbreaks (Netherer et al. 2015; Mezei et al. 2017). In addition to climate change, forest management has a substantial impact on the susceptibility of forests to damage from a wide range of disturbance agents (Seidl et al. 2011; Senf and Seidl 2021). Forest adaptation strategies modify and shape the development of forest characteristics and species composition to achieve specific goals. To increase the resilience of managed forests, consideration needs to be given to how climate change affects forest development as well as what characteristics influence forest susceptibility to damage from both abiotic and biotic disturbance agents (Girona et al. 2023). Intensively managed even-aged forest stands tend to contain many trees of the same species and age, which provide abundant resources that some insect pests can take advantage of (Kärvemo et al. 2014; Mezei et al. 2014; Hlasny et al. 2021). To enhance the resilience of such intensively managed stands while simultaneously balancing ecological and economic goals, additional consideration needs to be given as to whether this is achieved by enhancing natural ecological processes (e.g. via increased forest compositional or structural complexity) or via repeated intensive interventions (e.g. shorter rotations, variable intensity thinnings or patch cuttings) (Čada et al. 2016; Felton et al. 2024b). In this study, the term disturbance is used in a broader applied sense to include both acute and chronic factors that significantly influence forest dynamics, and productivity or management outcomes over relevant time scales.

In recent years, Nordic forests have recorded increasing damage trends from some biotic and abiotic disturbances (San-Miguel-Ayanz et al. 2021; KSLA 2022; Skogsstyrelsen 2022; Patacca et al. 2023; Knutzen et al. 2025). In the last ten years, Norway has registered unusual drought periods and climate models project additional changes in patterns of precipitation, with increased annual precipitation likely to elevate the risk of flooding and heavy rainfalls (Hanssen-Bauer et al. 2017). In addition, Finland has observed increases in damage due to drought, bark beetles and wind since the year 2018 (Neuvonen 2020). These events have led to a growing interest in alternative ways to manage forests to limit or prevent these types of major damage events. In Sweden, forest management for high yield wood production often leads to forests dominated by single species like Norway spruce (*Picea abies* (L.) H. Karst.) or Scots pine (*Pinus sylvestris* L.) with associated homogeneous forest characteristics typically including a small proportion of other conifer and broadleaf tree species. These homogeneous conditions contribute to the forest’s susceptibility to damage by biotic agents specialized to exploit the tree species and age structures provided (Guyot et al. 2016; Huuskonen et al. 2021). For example, at present about 80% of the forest land in Sweden is managed for wood production, with these forests limited to “productive” forest land, i.e. capable of producing ≥ 1 m^3^ of wood ha^−1^ yr^−1^ (SLU 2025).

Currently, a range of available practices are considered useful for the adaptation of production orientated forests to major disturbance. However, many of these management practices focus on the management of one specific disturbance. As much of the research on the adaptation of forests to specific disturbances is done in isolation, this review is an attempt to bring together and provide an overview of different adaptation strategies and their expected consequences for individual disturbances, with the aim to find synergistic, neutral, or antagonistic responses of adaptation strategies for the disturbances included in the study.

The objectives of our literature review are: (1) to identify how forest characteristics and tree species composition affect forest susceptibility to damage caused by ten major disturbance agents, and (2) to assess the effectiveness of seven adaptation strategies at reducing forest susceptibility to these major disturbance agents in Sweden. In the review, we define forest adaptation strategies as silvicultural practices for controlling and directing forestry to achieve some predetermined values or characteristics at the stand level. We mainly focused on even-aged Norway spruce or Scots pine dominated production-orientated forests since approximately 82% of the productive forest land in Sweden is dominated by them and are often grown in even-aged single tree species plantations (SLU 2025). The seven adaptation strategies covered are all well known by forest companies and smaller-scale private forest owners: mixed-species stands, shorter rotation periods, longer rotation periods, few or no thinnings, logging residue removal, prescribed burning, and uneven-aged forestry (selection felling and patch cutting). We evaluated the influence of these strategies on forest susceptibility to ten major natural disturbances in Sweden covering weather-related damage, insect damage, pathogen damage and browsing damage. Although the primary motivation of our study resulted from challenges facing Sweden, the review encompasses literature from multiple countries worldwide, with a focus on boreal forests, particularly those dominated by Norway spruce and Scots pine. In addition, the natural disturbances we target are commonly found in Sweden’s forests and are well known internationally. Similarly, the management adaptation strategies we explore enhance the relevance and applicability of our study findings across various international contexts.

## Materials and methods

The research was conducted through a mixed methods approach, by using knowledge gathered during an online workshop with experts in forest damage, in combination with a literature review. The online workshop facilitated knowledge sharing, asking questions, and hearing different perspectives, which was important given the ten different disturbance types covered by the review, many of which require different management approaches. In addition, it served as a good basis to discuss and reach a consensus on the scope of the review.

### Workshop

#### Identification of disturbance agents and forest adaptation strategies

Fourteen researchers studying different aspects of forests ecology, including forest-dwelling species and forest disturbance agents and an advisor from the Swedish Forest Agency participated in the full-day workshop on the 31st of March 2022, to share their knowledge and expertise on disturbances and potential adaptive silvicultural practices to reduce damage in the forest. Before the workshop, each expert was given the task of identifying the most significant disturbances from an economic perspective and prepare a list of management suggestions to reduce the damage caused by each disturbance. The participants presented a major category of disturbance on which they had expertise, which was followed by an interactive discussion that explored potentially effective management strategies for their respective categories of disturbance. A key outcome of the workshop was to provide participants with a comprehensive view of the management options available that may reduce damage, as well as insights regarding potential synergistic benefits of such strategies among the different categories of disturbance assessed. The discussions were directed toward the provision of a definitive list of disturbances to include in our review, based on their perceived importance to stakeholders and the time availability for the review’s completion. We also agreed on the study's objectives, as well as the inclusion and exclusion criteria outlined in the “Literature Search” section.

In total, ten natural disturbance agents (two abiotic, eight biotic) were selected based on their relevance and economic importance in Sweden. Two of the most relevant weather-related disturbances in Sweden, wind and snow damage, were included in the review. Three insect species that are known to be important source of forest damage in Sweden were selected: (1) the European spruce bark beetle (*Ips typographus*, L.; Coleoptera; Curculionidae), a specialist on Norway spruce that attacks mature spruce trees (Göthlin et al. 2000); (2) the European pine sawfly (*Neodiprion sertifer* Goeffroy, 1785; Hymenoptera; Symphytae), a species with semi-regular outbreak dynamics and a specialist on pine (*Pinus* spp.); (3) and the Large pine weevil (*Hylobius abietis*, L; Coleoptera: Curculionidae), a generalist herbivore and a pest in newly planted forest stands. The European pine sawfly was chosen for this study over other defoliators in Sweden, such as the nun moth (*Lymantria monacha*) or the pine beauty (*Panolis flammea*), due to its history of consistently exhibiting outbreaks over the decades. In relation to pathogens, four species known for causing important damage in Sweden were selected in this review: two species, known to cause root rot, from the genus *Heterobasidion* spp., *Heterobasidion annosum* sensu lato (Fr.) Bref., which infects a wide range of tree species, and *Heterobasidion parviporum* Niemelä and Korhonen, which predominantly targets Norway spruce; *Armillaria* spp., a species that causes root rot and infects a variety of conifer and deciduous trees and shrubs; the Scots pine blister rust caused by the *Cronartium pini* which specifically affects Scots pine and is showing an increasing incidence of damage in Sweden. The final natural disturbance covered was browsing damage by large-bodied herbivores (for example, moose (*Alces alces*), roe deer (*Capreolus capreolus*), red deer (*Cervus elaphus*), and fallow deer (*Dama dama*)). Browsing damage can appear in two main ways: firstly, as damage to small regenerating trees (top shoot browsing) and secondly, as damage to large mature trees, typically in the form of bark damage. This review primarily addresses the former, as damage to regeneration poses a greater challenge in production forests.

We chose seven adaptation strategies to include in this review based on (1) the possibility of applying the strategy at the stand level, (2) the potential of each strategy to influence the forest's susceptibility to damage by various disturbances, and (3) what strategies are most used to tackle disturbance damage in Sweden. We used a reference ‘baseline strategy’ for comparison with the seven adaptation strategies. The baseline strategy consisted of single tree species plantations of Scots pine or Norway spruce, in which a precommercial thinning and two to three thinnings are performed, and final felling is conducted at the most economically advantageous rotation time. Precommercial thinnings were assumed to be implemented five years after planting or when the forest is two to three meters high. Commercial thinnings are performed several times during the rotation in stands between 15 and 50 years old. The adaptation strategies selected for this review as well as the baseline strategy are described in Table 1.

**Table 1:** Description of the adaptation strategies included in the review. The “Baseline” strategy is described on top followed by the description of the seven adaptation strategies

Adaptation strategy	Description
Baseline (business as usual)	Planting of Scots pine or Norway spruce, Performing a precommercial thinning,
	Two to three thinnings, and
	Final felling at the most economically advantageous rotation time
Mixed-species stands	Same as baseline but:
	A maximum of 70% of basal area is allowed for the dominant species, and
	Natural regeneration is allowed
Shorter rotation period	Same as baseline but:
	Final felling is done before the most economically advantageous age is reached
Longer rotation period	Same as baseline but:
	Final felling is done after the most economically advantageous age is reached
Few or no thinnings	Same as baseline but:
	At most only one thinning is performed during the rotation period
Logging residue removal	Same as baseline but:
	Logging residues (tree tops and branches) from thinnings and final fellings are removed from the forest, and/or
	Tree stump removal (or tree stump treatment) in connection to root rot due to its special relevance to this disturbance
Prescribed burning	Same as baseline but:
	Performing prescribed burns to favour the presence of alternative species in the understory, and/or
	Prescribed burning can also be applied for site preparation after final felling
Uneven-aged forestry	Selection felling involves the harvesting of trees of different ages and sizes to generate an uneven-aged structure (an approximate inverted J-shaped stem diameter distribution) in a forest stand over time
	Patch cutting involves the harvesting of a forest area of at most 0.25 ha to maintain a continuous cover over time and generate an uneven-aged structure in the forest

### Literature search

The search of publications was restricted to the English language, and from the year 2000 to the date of the literature search, which was conducted from the 5th of September 2022 to the 20th of February 2023. The search was restricted to this period to review modern approaches to adapting forests to natural disturbances. To obtain comprehensive results, all academic citation databases included in Web of Science (WoS) as well as SCOPUS database were used to search for publications.

Each search paired a disturbance-related string and an adaptation strategy-related string, along with a constant string for study population and geography. To cover all disturbances thoroughly, six search strings were used, as were six for adaptation strategies. Hence, each search comprised three strings: study population/geography (unchanged) (string 1), a disturbance (string 2), and an adaptation strategy (string 3). Importantly, each search combined these strings uniquely, matching the total number of possible combinations between disturbances and adaptation strategies. This led to 36 searches in Web of Science and another 36 in SCOPUS (see supplementary material S1 for more details). A brief description of the three strings per search is as follows:

1. String 1: Study population and geography. The formulated string comprises literature associated with the two most important tree species in Sweden from an economic point of view, *Pinus sylvestris* and *Picea abies*. Likewise, literature associated with the boreal and temperate regions around the world is also covered.
2. String 2: Natural disturbance. One string per disturbance category (or individual disturbance agent) was formulated to capture associated literature.
3. String 3: Adaptation strategy. A unique string was built for each adaptation strategy except for lengthened and shortened rotations where only one string was used to catch publications for both lengthened and shortened rotations.

The search terms used were linked to each other by wildcards (*), quotation marks (“”), proximity operators (NEAR) and the connector ‘OR’. The search strings were connected to each other using the connector ‘AND’. The inclusion and exclusion criteria were based on identifying, through a first title and abstract screening, the articles that could potentially respond to the stated objectives of the study. The Rayyan tool (Rayyan.ai) was used for the first screening of the articles. The extraction of information from the articles was done systematically and based on a protocol that consisted of several standardized tables to systematically extract and organize relevant data from each article. During the extraction of information from each article, each of the experts was asked to evaluate the quality of the studies reviewed. For this purpose, a specific column was added to the protocol where this information was collected. In total, 2318 unique search hits were obtained from the search and loaded into Rayyan tool for screening. Authors accessed Rayyan to decide on inclusion or exclusion of articles based on title and abstract. The number of articles discarded in this step was of 2110. The remaining 208 articles were read in detail. The final number of peer-reviewed articles that were selected from the search and included in the review was 87.

In addition to the papers found within the search, additional peer-reviewed articles and reports that were considered relevant for the purpose of this review were included without regard to time of publication. This was done to ensure that relevant literature for this study would be included. Variation in the terminology used in the literature for the same management intervention may have influenced the comprehensiveness of our search, and thus whether some articles were missed. To mitigate the risk of excluding important literature, the experts involved were given the freedom to include any literature they deemed relevant that was not found in the initial search. In total, 45 articles were added to the review for completeness of the search. Supplementary material S1 gives a detailed description of the search terms employed for every feature. Supplementary material S2 presents a list of the articles found in the search as well as those additional articles provided to complement the review.

## Results

### Forest characteristics

**Fig. 1:**
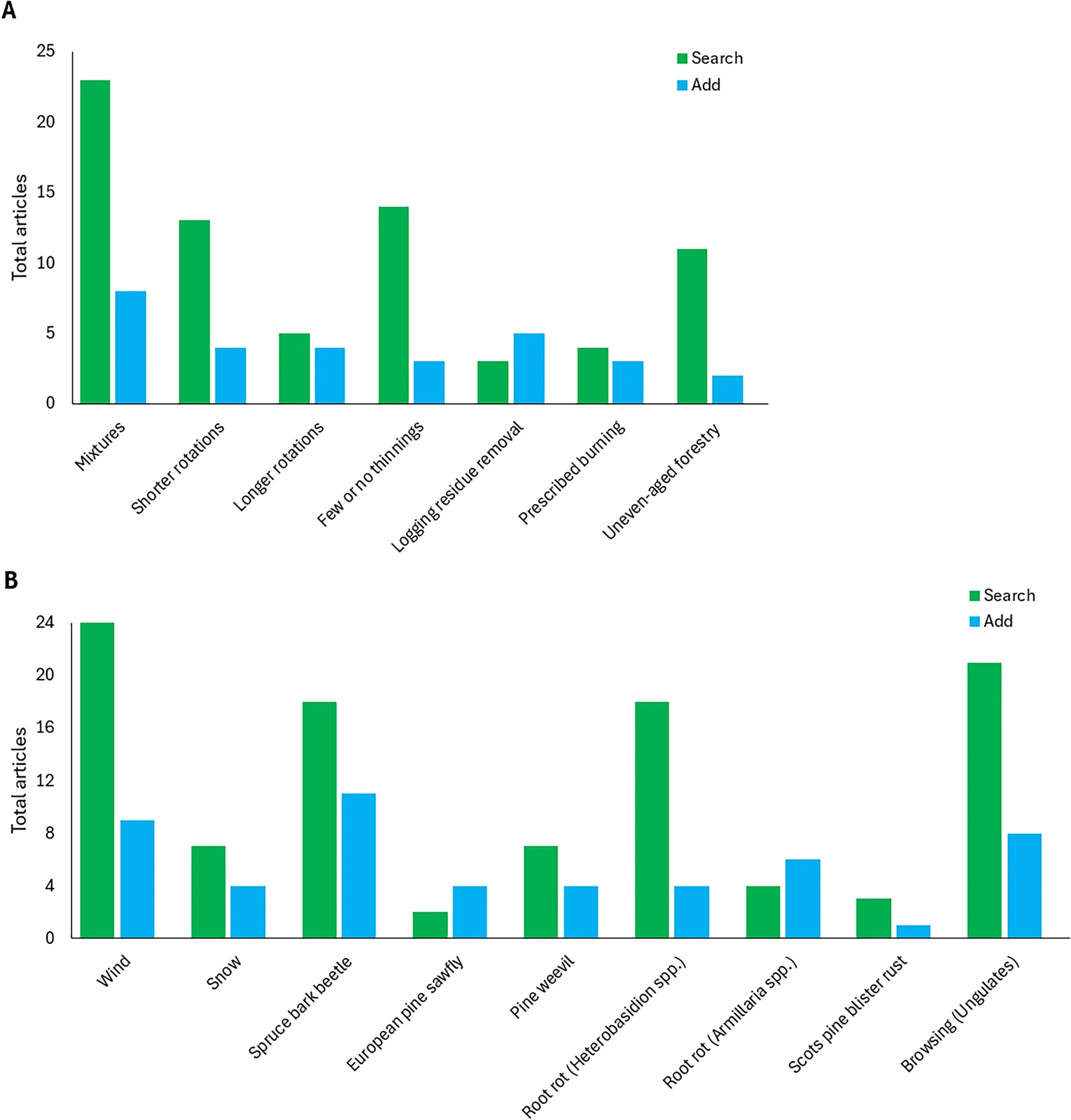
Number of studies per adaptation strategy (**A**) and disturbance agent (**B**). The green columns show the number of articles that were found within the search, and the blue columns show the additional articles that were added to the review to complement the ones found in the original search. Note that an article may be included in several disturbance columns and adaptation strategies

This section describes each disturbance category reviewed in relation to forest characteristics and tree species compositions that differentially affect forest susceptibility to the disturbance. Figure 1 presents the total number of articles found in the literature search and the additional ones that were added to the review as a supplement to first ones.

### Weather-related damage

#### Wind damage

The risk of wind damage in forests is highest in mature stands with tall trees (e.g. Gardiner et al. 2013; Jönsson et al. 2015; Díaz-Yáñez 2018). In general, conifers are more sensitive to wind compared to broadleaves (Schmidt et al. 2010; Albrecht et al. 2012). Evergreen conifers are more susceptible to winter storms as foliage can exacerbate wind drag during storms and increase the likelihood of breakage or uprooting (Päätalo et al. 1999; Valinger and Fridman 2011). Differences in crown and rooting characteristics between tree species can influence the susceptibility to wind damage (Wohlgemuth et al. 2022). The height-to-diameter ratio (taper) is an important parameter for susceptibility where trees with low taper tend to have higher individual stability (Wonn and O’Hara 2001; Mason 2002; Díaz-Yáñez et al. 2017b). Norway spruce appears to be more affected by wind damage compared to other tree species (Schütz et al. 2006; Gardiner et al. 2013). Replacing or promoting admixtures of Norway spruce with other species, such as birch (*Betula* spp.), hybrid aspen (*Populus tremula* L. × P*. tremuloides* Michx*.*), Douglas fir (*Pseudotsuga menziesii*), or other less vulnerable tree species, can thus decrease damage risk (Valinger and Fridman 2011; Subramanian et al. 2016, 2019; Ruel 2020; Fuchs et al. 2022). Other factors, such as wind exposure of trees (forest gaps and edges) or the state and type of soil, also affect forest susceptibility to wind and need to be considered (Roberts et al. 2007; Pasztor et al. 2015; Donis et al. 2018). In addition, increasing the spread of the age class distribution and therefore promoting a range of tree sizes can decrease wind damage (Pukkala et al. 2016). Conversely, other silvicultural practices and forest characteristics, such as the removal of large basal areas during commercial thinnings and the creation of new forest edges by the harvesting of neighbouring forests greatly increases tree exposure to wind and thus the susceptibility to wind damage of the forest area connected to the edge (Valinger and Pettersson 1996; Zeng et al. 2006; Vyse et al. 2008; Anyomi and Ruel 2015; Gardiner et al. 2016).

#### Snow damage

Stand density, stand age, and tree species are the most important parameters determining susceptibility to snow damage (Valinger and Pettersson 1996; Päätalo et al. 1999; Díaz-Yáñez et al. 2017b, 2019). In stands with high tree densities, snow interception is higher, and this accumulation of snow could lead to increased top and branch breakage, especially in evergreen tree species (Hedstrom and Pomeroy 1998; Wonn and O’Hara 2001; Vyse et al. 2008; Díaz-Yáñez 2018).

### Insect damage

#### Spruce bark beetle damage

The European spruce bark beetle is a pest of mature Norway spruce stands throughout Europe (Christiansen and Bakke 1988; Netherer and Nopp-Mayr 2005). At endemic levels, spruce bark beetles attack weakened or dying spruce trees (Hroššo et al. 2020), whereas at epidemic levels the beetles are also capable of attacking healthy trees (Wermelinger 2004). After large wind fellings, with beneficial weather conditions the beetles can rapidly increase their population densities. Trees weakened during prolonged periods of drought also have heightened susceptibility to spruce bark beetle (Wermelinger 2004; Mezei et al. 2017). After the start of an outbreak (epidemic densities) their dispersal in the landscape and subsequent damage is related to forest characteristics and the continuity of host tree species in the forest (Klapwijk et al. 2016; Seidl et al. 2016; Dobor et al. 2020). The volume of spruce in the landscape is one of the most important determinants for subsequent damage levels (Kärvemo et al. 2014, 2016), together with the prevalence of mature trees with large stem diameters (Göthlin et al. 2000). The presence of non-host tree species, especially birch, appears to deter high levels of attack through natural dilution effects (Zhang and Schlyter 2004; Seidl et al. 2008; Kärvemo et al. 2016), but damage is not completely deterred, and the magnitude of the damage may be attributable to other factors than tree diversity (Faccoli and Bernardinelli 2014; Berthelot et al. 2021; Müller et al. 2022; de Groot et al. 2023).

#### European pine sawfly

European pine sawfly dynamics are thought to be more closely related to natural enemy pressure than to forest stand characteristics (Bellone et al. 2017). However, there are indications that increasing tree species diversity and reducing tree density could reduce resources for sawfly reproduction (Veteli et al. 2006; Kaitaniemi et al. 2007; Klapwijk and Björkman 2018). Sawfly population dynamics are thought to also be indirectly affected by forest characteristics which alter the prevalence of natural enemies (Klapwijk et al. 2016). Local structural complexity in forest stands appears to be a more important influence on for predation level by arthropod predators than stand level complexity (Bellone et al. 2020, 2021). Cocoon predation by small mammals seems to be more influenced by local heterogeneity in the form of dead wood and tree density than by other stand characteristics (Bellone et al. 2017).

#### Pine weevil

The large pine weevil causes high levels of damage in newly regenerated forests. This damage occurs when stumps from harvested trees are left on recent clearcuts. The adults swarm into clearcuts in response to the high volatile emissions, lay their eggs on the tree stumps and feed on nearby food resources (Nordlander 1987). Later, the larvae develop in the ground on the roots of the stumps and will upon emergence feed on available food resources such as the bark of newly planted/regenerated seedlings (usually Norway spruce or Scots pine). Feeding often girdles the seedling, which leads to seedling mortality. Removing stumps and logging residues after harvesting might reduce the influx of adult weevils (Piri et al. 2020). Also, it has been proposed that increasing tree species diversity might reduce the damage on seedlings (Björkman et al. 2015; Klapwijk et al. 2016) but, so far, research has not provided conclusive evidence (Löf et al. 2004; Tudoran et al. 2021). In addition, the type of seedling, and whether it is naturally regenerated or planted, could affect the amount of seedling mortality (Selander et al. 1990). Prescribed burning may facilitate pine weevil survival and reproduction through the creation of favourable reproductive conditions such as less vegetation cover and darker colours contributing to higher temperatures on site (Hofsten and Weslien 2005; Pitkänen et al. 2008). As the densities of European pine weevil are maintained at a high level by the practice of clearcut harvesting which favours reproduction conditions, one way of reducing damage on seedlings is to avoid clearcuts and instead use multilayer forestry methods (Björkman et al. 2015; Klapwijk et al. 2016).

### Pathogen damage

#### Heterobasidion spp. root rot

*Heterobasidion* spp. enters trees and forest stands through newly created tree stumps and wounds on the surface of roots or lower parts of tree stems (Isomäki and Kallio 1974; Gunulf et al. 2013). These entry points are initially colonized by the fungus which can subsequently spread through the root system and across root contacts to infect new trees (Stenlid 1987). Avoiding the creation of new stumps and the application of treatment to fresh stumps with urea or a commercial formulation of the fungal biological control agent *Phlebiopsis gigantea* (Rotstop®) are the two most effective approaches to reduce infection and subsequent losses in both Norway spruce and Scots pine (Berglund and Rönnberg 2004; Oliva et al. 2010; Gunulf et al. 2012; Blomquist et al. 2020, 2023; Zaluma et al. 2021). Closeness to fresh stumps is also an important factor in infestation of healthy trees as is the proximity to strip roads due to potential injuries on tree roots or stem inflicted during harvesting operations (Metslaid et al. 2018). Prevention is essential for managing *Heterobasidion* spp. root rot because once the fungus infects a tree's root system in a stand, it becomes exceedingly challenging to remove or prevent its spread, even by aggressive treatments like stump removal (Cleary et al. 2013). In cases where stands are extensively infected, stump treatment may not yield a significant difference (Wang et al. 2015). Promoting tree species diversity has been shown to help reduce the spread of disease and associated damage to newly planted seedlings in some cases (Möykkynen and Pukkala 2011).

#### *Armillaria* spp. root disease

The development of Armillaria root disease is favoured by the availability of suitable substrates, including susceptible host trees and trees with reduced host vigour resulting from predisposing stresses. These conditions promote the establishment and spread of *Armillaria* species that function as either primary or secondary pathogens (Aslam and Magel 2018; Holuša et al. 2018). Tree stumps and root systems left behind after thinning or harvesting serve as long-term reservoirs of fungal inoculum (Cleary et al. 2013) thereby increasing the risk of infection to residual and regenerating trees through root-to-root contact or rhizomorph spread (Roberts et al. 2020). Selecting trees species that differ in susceptibility can reduce the overall impact of the disease (Cleary et al. 2021), however, this positive effect is not always observed across sites and stand conditions (Morrison et al. 2014; Čermák and Žid 2020). In addition to interspecific variation in susceptibility, tree species differ in rooting depth, architecture and spatial distribution, which can reduce the probability of pathogen transmission across root contacts compared with monoculture stands dominated by a single species (Cleary et al. 2008).

#### Scots pine blister rust

With respect to the literature reviewed and to the best of our knowledge, there are no connections between forest characteristics and species composition, and the susceptibility of forests to damage by Scots pine blister rust *Cronartium pini* (Kaitera 2002). The susceptibility to damage by *Cronartium pini* differs between pine species (Kaitera and Nuorteva 2008; Samils and Stenlid 2022), but among populations (provenances) of Scots pine, no differences in susceptibility have been demonstrated (Kaitera 2003; Kaitera and Nuorteva 2008).

### Browsing damage

Across Europe, many populations of large herbivores (here: different deer species) are increasing in numbers and expanding their range (Apollonio et al. 2010; Linnell et al. 2020), and their browsing pressure on economic valuable trees generates conflicts in managed forest landscapes (Côté et al. 2004; Beguin et al. 2016). Many of the studies reviewed in the literature highlight that tree species composition can greatly influence browsing pressure by deer species, and thus damage level (Edenius et al. 2015; Nevalainen et al. 2016; Champagne et al. 2021; Pfeffer et al. 2022). The diversity of tree species in the forest can influence forest susceptibility to browsing damage both positively and negatively (Díaz-Yáñez et al. 2017a). In addition, increased tree vigour has also been associated with higher incidence of browsing (Bergquist et al. 2003). At the stand scale, stands with greater tree species diversity may experience more browsing than stands dominated by single tree species with a lower diversity of palatable tree species (Vehviläinen and Koricheva 2006; Milligan and Koricheva 2013; Wallgren et al. 2013; Herfindal et al. 2015). Canopy openness and gap size are also important factors affecting browsing susceptibility. Forest clearings that receive a lot of light incur higher visitation rates by ungulates than closed patches, as there is more forage biomass available per unit space in the former (Kuijper et al. 2009). The density of trees in such regenerating clearings can however have a protective effect in relative terms, as it is often found that a high stem density is associated with a relatively low proportion of stems being damaged (Bergqvist et al. 2014; Pfeffer et al. 2021). Thus, changes in forage availability for feeding can lead to changes in browsing or herbivory pressure (Kramer et al. 2006; Loosen et al. 2021). At the landscape scale, an increasing amount of natural and attractive forage might become a key feature to reduce browsing pressure on pine in individual forest stands (Roberge et al. 2016; Felton et al. 2022b).

### Forest adaptation strategies

This section describes how the seven adaptation strategies can modify forest characteristics and tree species composition which in turn influence forest susceptibility to damage. These results are summarised in Tables 2 and 3.

### Mixed-species stands

Increasing species diversity in the forest contributes to spreading the risk from multiple damaging agents in production forests (Jönsson et al. 2015; Dobor et al. 2020; Honkaniemi et al. 2020) and is also beneficial for many ecosystem services (Felton et al. 2016, 2022a; Huuskonen et al. 2021). However, herbivores in general, and large browsers specifically, display selective foraging behaviour and therefore mixtures of different tree species might increase the chances of browsing damage at stand level (Vehviläinen and Koricheva 2006; Bergqvist et al. 2012, 2014; Milligan and Koricheva 2013; Nevalainen et al. 2016; Díaz-Yáñez et al. 2017a; Champagne et al. 2021). Ohse et al. (2017) observed that mixing Scots pine stands with seedlings of European ash (*Fraxinus excelsior*), silver birch (*B. pendula*), black alder (*Alnus glutinosa*), or Siberian larch (*Larix sibirica*) increases browsing likelihood. This phenomenon, known as associative susceptibility, suggests that forests with higher species diversity are more attractive to browsers compared to single tree species stands (Vehviläinen and Koricheva 2006; Herfindal et al. 2015). However, if less palatable tree species (e.g., Norway spruce) are included in the mixture, it may have the opposite effect and help reduce damage (Champagne et al. 2021). Mixtures of Norway spruce with wind resistant tree species such as Douglas fir (*Pseudotsuga menziesii* (Mirb.) Franco) can reduce susceptibility of the forest to damage by wind (Schütz et al. 2006; Fuchs et al. 2022). In general, tree species mixtures are thought to have lower susceptibility for wind damage (Dobor et al. 2020), which in addition would reduce the risk of spruce bark beetle outbreaks with wind damage as a cause (Jönsson et al. 2015). Conversely, the reduction of host tree availability for specialist species would reduce the potential infestation rates of pathogens and tentatively for pest insects (Lygis et al. 2004; Seidl et al. 2008; Möykkynen and Pukkala 2010; Faccoli and Bernardinelli 2014; Huuskonen et al. 2021). However, for polyphagous species (i.e. pests and pathogens with a broad host range) the choice, and proportion, of tree species in the mixture will be important where interspecific variation in susceptibility exists. On the other hand, mixed-species stands may promote wider spacing between trees as well as different rooting habits, thus lowering the probability of transmission across root contacts (Burdon and Chilvers 1982; Lindén and Vollbrecht 2002; Möykkynen and Pukkala 2011; Prospero and Cleary 2017). However, a study by Piri et al. (1990) found no significant correlation between tree species mixing and reduction in infection caused by *Heterobasidion* spp. Ultimately, mixed-species stands can be created in many ways. Hitherto, research has not sufficiently addressed the effects of different methods (scales) or combinations of mixing tree species in forest stands on their ability to mitigate damage risk from several agents simultaneously (Felton et al. 2022a).

### Shorter rotation periods

Harvesting trees at a younger age, i.e. shortening the rotation period, reduces the presence of mature trees and the damage caused by silvicultural practices like thinning, since fewer or no thinnings are performed. However, shorter rotations increase the frequency of final fellings and the density of tree stumps in the resulting clearcuts. High densities of tree stumps can attract pine weevils through increased attraction and simultaneously create additional infection courts by root pathogens. The proportion of mature forest exposed to wind damage would be reduced at the landscape level because of earlier harvests (Subramanian et al. 2016, 2019; Zimová et al. 2020). In addition, shorter rotations would reduce susceptibility to snow damage at the end of the rotation period by avoiding further exposure in time (Díaz-Yáñez 2018; Díaz-Yáñez et al. 2019). Shorter rotation periods are also an effective way to reduce the risk of spruce bark beetle damage due to reduced availability of suitable breeding materials such as large diameter Norway spruce trees (Overbeck and Schmidt 2012; Klapwijk et al. 2016; López-Andújar Fustel et al. 2024). Furthermore, early harvesting in infected stands may help reduce volume losses caused by root rot pathogens compared to delayed harvestings, which may exacerbate pathogen spreading along the stem base (Möykkynen et al. 2000; Subramanian et al. 2016). However, it is also argued that higher gains would not result from increased harvesting rates (Aza et al. 2021). On the other hand, pine weevil population densities and associated damage could increase with a higher frequency of harvesting and higher tree densities at harvest (Roberge et al. 2016; Doležal et al. 2021). In addition, young stands contain more forage available to herbivores than mature stands, if the latter provide high stem densities (Wam et al. 2010; Hedwall et al. 2013; Ara et al. 2022). Also, shortening rotation lengths may lessen bilberry cover leading to potentially higher browsing pressure on young stands in the regeneration phase (Roberge et al. 2016).

### Longer rotation periods

Mature stands are more susceptible to wind damage (Gardiner et al. 2013). If rotation periods are lengthened the number of thinning interventions is generally increased, which may lower the risk for snow interception (Díaz-Yáñez et al. 2019). However, harvesting trees later in the rotation increases the potential window of opportunity of infestation by insects and pathogens, for example, increasing the risk of spruce bark beetle attack in Norway spruce stands (Hlasny and Haas 2023). In addition, increasing the number of thinning interventions could increase the probability of root rot pathogens such as *Heterobasidion* spp. entering the stand with each stand entry operation, unless stumps are treated (Berglund and Rönnberg 2004; Möykkynen and Pukkala 2009). Lower harvesting frequency could also reduce pine weevil damage as well as new infestation rates by pathogens through reduced stump availability (Bendz-Hellgren and Stenlid 1998; Björkman et al. 2015). On the other hand, longer rotation times allow for more mature forests at the landscape scale, which can provide more forage for browsers in the field layer if a sufficient amount of light reaches the forest floor (Felton et al. 2020b).

### Few or no thinnings

Reducing or avoiding thinnings will reduce mechanical damage to the trees that can occur during these operations and serve as an entry point for pathogens such as *Heterobasidion* spp. (Mäkinen et al. 2007). In addition, fewer or no thinnings reduce the availability of freshly cut stumps that can be colonized by root rot fungi such as *Heterobasidion* spp. or *Armillaria* spp. species which would otherwise increase the inoculum potential of the fungi, thereby limiting inoculum build-up and the subsequent risk of infection to residual or regenerating trees (Rönnberg et al. 2006, 2013; Gunulf et al. 2013; Wang et al. 2015; Morrison et al. 2022). When combined with lower planting densities, limiting the number of thinnings can also reduce the need for future forest operations and lower the risk of wind damage (Subramanian et al. 2019; Samariks et al. 2020). In addition, the absence of thinnings would avoid creating canopy openings and the loss of support between neighbouring trees which could otherwise increase the risk of windthrow (Valinger and Pettersson 1996; Hahn et al. 2021), although this is not always the case (Vyse et al. 2008). In contrast, if initial planting densities are not lowered, fewer thinnings will result in higher tree densities and potentially less vigorous trees; as the higher tree densities increase competition for resources between trees in the stand, and the least vigorous trees that are often removed during thinnings will remain in the stand. A reduction in tree vigour might increase the susceptibility of the forest to insect pests such as spruce bark beetle (Klapwijk et al. 2016). A closed canopy will not allow the sunlight to reach the understory, decreasing the chances of natural regeneration or the presence of vegetation under the main canopy (Hedwall et al. 2013). In addition, a high Scots pine tree density has been related to a higher susceptibility of the forest to the European pine sawfly damage (Veteli et al. 2006). However, greater competition for the available resources might lead to a greater presence of deadwood in the forest from self-pruning and death of suppressed trees. On the other hand, precommercial thinnings aiming to remove competition and thereby help pine trees quickly grow out of browsing height, aids in reducing browsing damage (Härkönen et al. 2008; Wallgren et al. 2013; Ara et al. 2022). Furthermore, already browsed trees should be left standing as they are likely to be re-browsed and therefore reduce the risk of a previously undamaged pine tree being damaged instead (Bergqvist et al. 2001; Wallgren et al. 2013).

### Logging residue removal

Leaving or removing logging residues from the forest does not appear to have any influence on populations of spruce bark beetle except if large diameter logging residues are removed (Thiel 1999; Eriksson et al. 2006). Conversely, leaving logging residues in the forest has been associated with higher pine weevil damage, especially in the surroundings of residue piles (Hanssen et al. 2017; López-Villamor et al. 2019). In relation to browsing damage, leaving logging residues in the forest may increase the availability of forage (and thereby decrease the risk of damage to the production stems), however, its use by ungulates might be influenced by the time since harvesting and the period of the year (Edenius et al. 2014). In this context, Loosen et al. (2021) observed that leaving enough biomass on slash piles may lead to lowered browsing pressure in nearby forest stands. To our knowledge, there are no studies of the effect of logging residue removal on ungulate browsing pressure. The removal of stumps and other residues may contribute to reduce pine weevil damage in the subsequently regenerating forest (Piri et al. 2020). In addition, the removal of stumps and roots can reduce the entry points for *Heterobasidion* spp. spores establishing on fresh stumps in new clearcut areas, however the physical removal of inoculum is less effective for controlling *Heterobasidion* root rot than for *Armillaria* (Cleary et al. 2013).

### Prescribed burning

Depending on the fire intensity, prescribed burning could change tree bark properties, reduce the water availability, and alter the composition of the phloem affecting the quality of resources used by some insect pests such as spruce bark beetle (Toivanen et al. 2009; Hekkala et al. 2021). In addition, natural predators of spruce bark beetle, for example *Thanasimus* spp., might be favoured by the application of prescribed burning (Hekkala et al. 2021). Conversely, pine weevil populations are attracted to burned areas and higher regeneration damage is recorded nearby (Pitkänen et al. 2008). In addition, prescribed burning does not appear to have an effect on root rot pathogens (including those investigated in this study), nor on browsing damage (Whitney and Irwin 2005; Lygis et al. 2010; Fredriksson et al. 2023). However, Hardenbol et al. (2021) did observe an effect on browsing after prescribed burning, suggesting that it may alter the palatability of tree species and thereby reduce damage.

### Uneven-aged forestry

Multilayered forests can be created in various ways. Here, patch and selective cuttings are considered. In patch cuttings small areas (up to 0.25 ha) can be harvested creating small clearcuts. In selective cuttings individual trees are singled out for harvesting, avoiding clearcuts and creating forests with full canopies. Uneven-aged forests often have lower proportions of timber exposed to wind which in turn might lower susceptibility to windthrow (Hanewinkel et al. 2014; Pukkala et al. 2016). The absence of clearcuts prevents the creation of new forest edges which contributes to maintaining canopy support between neighbouring trees and thus making forests less susceptible to wind damage (Pukkala et al. 2016). In contrast, Potterf et al. (2022) argues that high numbers of selective cuttings to keep the continuous cover and the tall dominant trees present in the forest might increase the susceptibility to wind damage. In addition, forest gaps located in wind exposed areas can incur greater wind damage (Roberts et al. 2007). In relation to browsing damage, patch cuttings and selectively logged sites might be especially attractive for ungulates due to tree regeneration in open areas (Kuijper et al. 2009; Atte et al. 2020). On the other hand, if natural regeneration is favoured in patch cuttings, the vegetation of shrubs and herbs can be dense and act as a natural barrier for browsing, reducing the overall damage to the tree seedlings (Walters et al. 2016). Furthermore, selective cuttings have been linked to an increased probability of infection caused by Armillaria spp., where tree diameter is the most important factor influencing the magnitude of the infection (Morrison et al. 2001). The implementation of repeated selective cuttings can contribute to generating more mechanical damage, which in turn increases the risk of new pathogen infections (Nevalainen 2017; Metslaid et al. 2018). Furthermore, uneven-aged forests with variation in age structure also changes the availability of suitable host trees, and for instance, these forests might be less affected by spruce bark beetle damage than even-aged stands (Klapwijk et al. 2016; Mohr et al. 2024). However, Seidl et al. (2008) argues that this strategy may lead to forests with high susceptibility to spruce bark beetle attack unless other measures are applied jointly such as promoting mixtures with non-host tree species. Research on multilayer forests is sparse, hence, caution with discussion of potential effects is required.

**Table 2:**
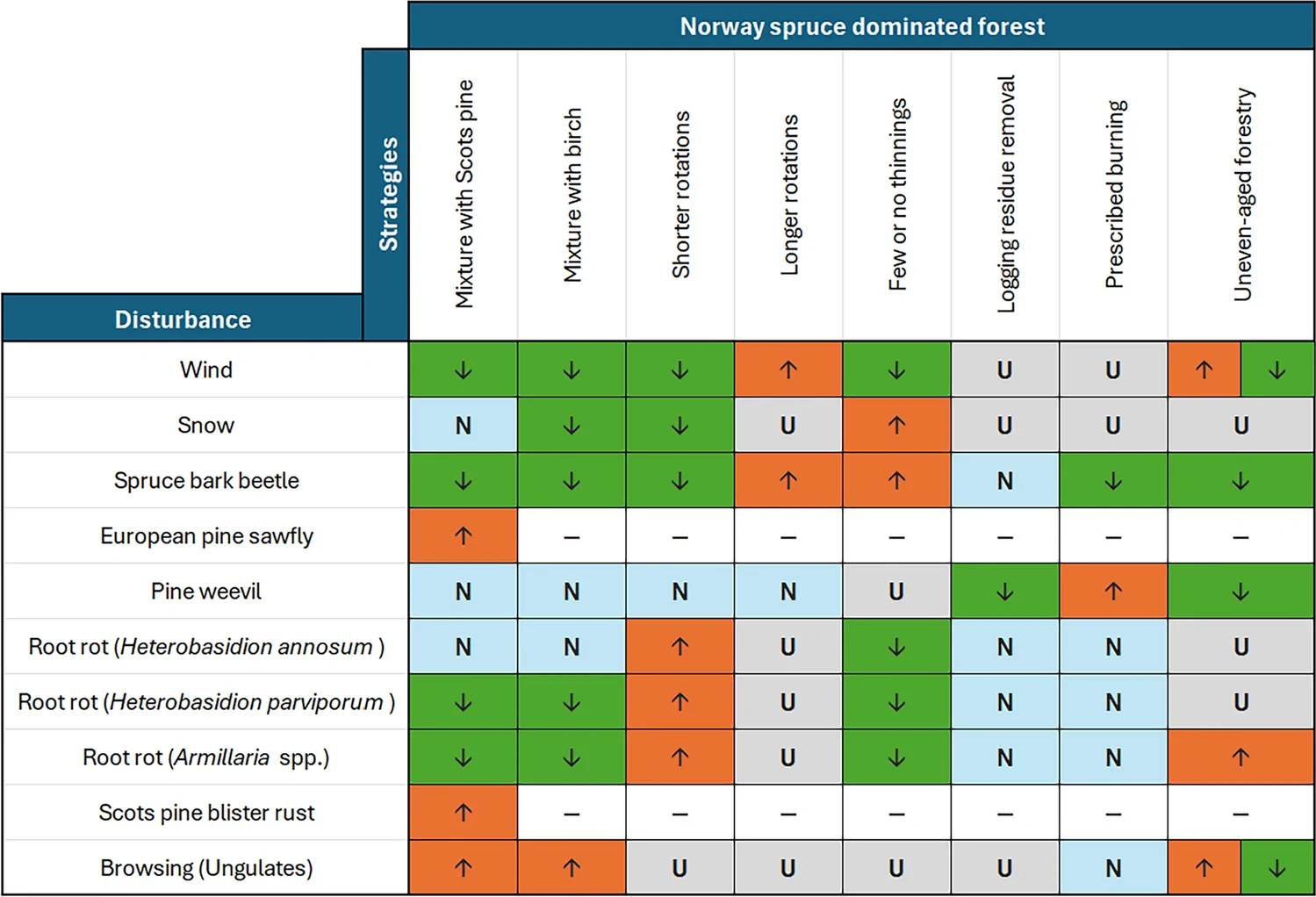
The matrix shows the direction of impact of each of the adaptation strategies on forest susceptibility to each disturbance agent for a Norway spruce dominated forest. An upward arrow means that the forest stand will become more susceptible to damage, a downward arrow means that the forest will become less susceptible to damage, if both types of arrows are shown the adaptation strategy can both increase and decrease the susceptibility to damage by influencing different forest characteristics, the letter “N” means that the effect of an adaptation strategy is neutral, and the letter “U” means that the effect of an adaptation strategy on forest susceptibility is unknown, the character “− “ is shown if the disturbance agent does not apply to a Norway spruce or Scots pine dominated forest

**Table 3:**
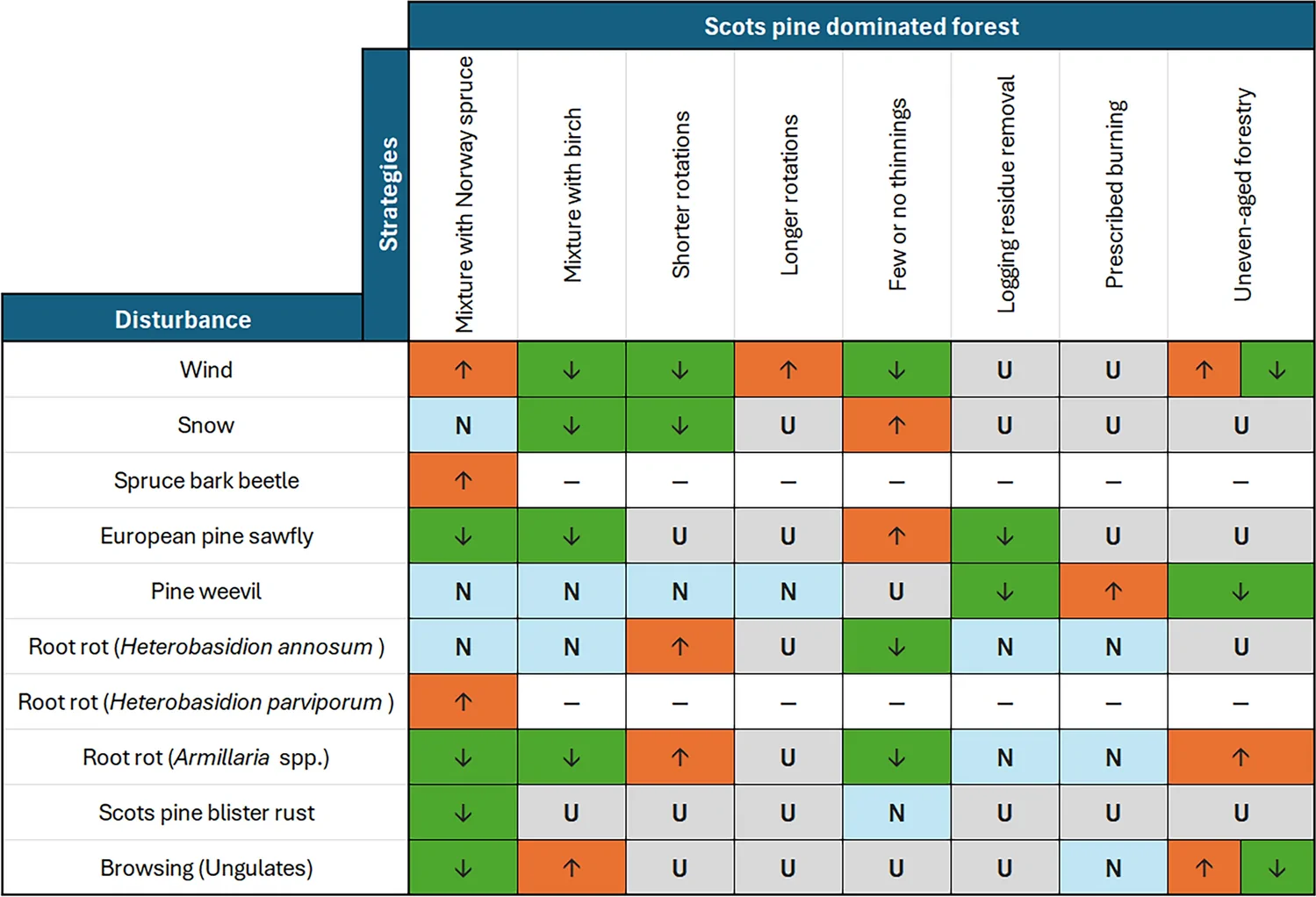
The matrix shows the direction of impact of each of the adaptation strategies on forest susceptibility to each disturbance agent for a Scots pine dominated forest. An upward arrow means that the forest stand will become more susceptible to damage, a downward arrow means that the forest will become less susceptible to damage, if both types of arrows are shown the adaptation strategy can both increase and decrease the susceptibility to damage by influencing different forest characteristics, the letter “N” means that the effect of an adaptation strategy is neutral, and the letter “U” means that the effect of an adaptation strategy on forest susceptibility is unknown, the character “− “ is shown if the disturbance agent does not apply to a Norway spruce or Scots pine dominated forest

## Discussion

Our literature review highlights the potential for adaptation strategies to exert influence over forest characteristics and species composition that are most relevant to decreasing forest susceptibility to different disturbance agents simultaneously.

### Adaptation strategies and natural disturbances

Even though the implications of a single adaptation strategy on forest susceptibility varied largely among disturbance agents, several patterns can be discerned. In line with Felton et al. (2024a) and Čada (2016), our review shows that strategies promoting diversity in forest characteristics, increased tree species richness, and fewer interventions during the rotation period tend to reduce forest susceptibility to several of the disturbance agents considered. Promoting both tree species and structural diversity in boreal forests may enhance the forests' ability to withstand and recover from natural disturbances. The importance of tree species diversity for forest resilience has been advocated not only for the tree species addressed in our study, but also for other boreal systems, such as black spruce (*Picea mariana* (Mill.)) and trembling aspen (*Populus tremuloides* Michx.) in Canada (Chavardès et al. 2021), as well as deciduous admixtures in Finland (Domisch et al. 2024). On the other hand, effective adaptation strategies for reducing forest susceptibility to a specific disturbance should focus on targeting key forest characteristics that link forest susceptibility to that particular disturbance. For instance, understanding the resource needs of biotic disturbances, which have varying life cycles, helps in selecting adaptation strategies to effectively modify forest resources and create less favourable conditions for these agents (Björkman et al. 2015; Marini et al. 2022).

Mixed-species stands may adversely affect specialist insect species while enhancing resources for polyphagous pest species (Koricheva et al. 2006; Jactel et al. 2021). However, forest stands with greater tree species diversity often experience higher rates of browsing damage (Bjørneraas et al. 2012; Felton et al. 2016), since large herbivores require a diverse range of tree species or bushes to fulfil their dietary needs. Implementing shorter rotations can effectively decrease exposure to some disturbances such as wind, reducing the likelihood of damage compared to forests left unharvested for longer periods (Subramanian et al. 2016; Zimová et al. 2020). This is because tall, large-diameter trees with extensive root systems, and big canopies, are more susceptible to damage from pests like spruce bark beetle and root rot pathogens (Netherer and Nopp-Mayr 2005; Gunulf et al. 2013). In addition, reducing the total number of thinnings throughout the rotation can help preserve specific forest characteristics like stem density, which decreases the risk of wind damage as the forest matures. Uneven-aged forestry offers an alternative to rotation forestry as a means of modifying forest characteristics, affecting the availability of young and mature trees, and in turn the forest susceptibility to some agents such as pest outbreaks and fungal pathogens (Morrison et al. 2001; Pukkala et al. 2016; Nevalainen 2017). The empirical relationship between this strategy and various agents remains largely unexplored, leading to uncertainty about its full potential (Hanewinkel et al. 2014). In relation to the longer rotation strategy, the literature reviewed in this study indicates that, at the stand level, it does not provide clear benefits in reducing forests' susceptibility to natural disturbances. However, longer rotations are frequently associated with enhanced biodiversity, highlighting additional considerations that, while not covered in this review, warrant acknowledgment. In addition, implementing longer rotations at larger spatial scales, such as at the landscape level, can reduce the forests' susceptibility to certain damage agents, such as that caused by pine weevil. Overall, our results highlight the complexity of outcomes that can result from management strategies employed to mitigate forest disturbances.

Trade-offs frequently emerge whereby the same management strategy can result in positive outcomes for some disturbance agents while increasing the vulnerability of forests to others. For example, converting single-tree species stands of Norway spruce into mixtures involving Scots pine generally increases resistance against wind damage, spruce bark beetle, and certain pathogens (e.g. *Heterobasidion parviporum* and *Armillaria* spp.). However, this strategy may also increase stand susceptibility to the European pine sawfly or browsing damage. Similar conflicts arise with shorter rotation periods and few or no thinning strategies. Given these conflicts, management decisions should consider the forest's geographical location, the dominant disturbance agents in the area, and the forest owners’ objectives and preferences. For example, optimal management practices for different forests within the same geographical region, with similar characteristics and facing similar risks may vary according to specific objectives and preferences of forest owners. Moreover, up-to-date information about climate change trends and projections for specific forest regions may be essential to ensuring evidence-based management decisions. A combination of different management strategies (e.g. mixed-species stands and changed rotation periods in the same forest stand) is also a viable option; one that this review has not fully explored. In this regard our findings indicate that implementing multiple strategies simultaneously may help reduce the risks posed by various disturbances more effectively than applying strategies in isolation.

Interactions among natural disturbances play an important role in altering forest development and susceptibility to damage (Ruel et al. 2023). The impact of one disturbance can alter or change forest conditions and thereby affect the susceptibility of the forest to other disturbances (Buma 2015; Venäläinen et al. 2020). Wind damage, for example, may precede insect outbreaks such as spruce bark beetle outbreaks (Lindelöw and Schroeder 2008). In addition, abiotic disturbances such as wind damage can weaken tree health by causing stem or branch breakage, making them more prone to pathogen infections and vice versa (Dale et al. 2016; Gaitnieks et al. 2021). *Armillaria* spp., including those typically known as secondary pathogens, can change their behaviour to become opportunistic on hosts that have reduced vigour and compromised defences as a result of other stress factors, potentially accelerating tree mortality (Kubiak et al. 2017). These interactions between disturbances can expand the area of susceptible forest, increase potential damage, decrease resilience and thereby prolong recovery times. Management planning should therefore consider these interactions, as adaptation strategies might have cascading effects modifying forest susceptibility to natural disturbances differently.

### Spatial scales on disturbance management

This review primarily focuses on stand-level management, yet forest management extends to broader scales with implications for diverse forest ownership and fragmentation. Notably, most disturbance agents affect forests beyond the stand level, resulting in a spatial mismatch applied in management (Beguin et al. 2016). The implementation of adaptation strategies to reduce susceptibility at larger scales is challenged by varying objectives of forest owners. Adaptation strategies at the landscape level influence forest susceptibility to disturbance agents, though research linking landscape management to damage remains limited (Felton et al. 2020a). Wind exposure, determined by the exposed canopy surface, underscores the importance of landscape-level silviculture in altering these characteristics (Gardiner et al. 2013; Donis et al. 2018). For specialist insect pests like the spruce bark beetle, concentrated host tree species or forest characteristics at landscape level can cause beetle populations to grow to epidemic levels and cause important impacts over vast forest areas. Conversely, when the resources required by these pests are dispersed throughout the forest landscape, the impact on vegetation is much smaller (Hlasny et al. 2019). Furthermore, the landscape scale is important for pathogens like *Heterobasidion* spp., which depend on freshly cut stumps or wounds on tree bark to spread and colonize new forest areas (Isomäki and Kallio 1974). Once these pathogens are established on a site, eradication becomes nearly impossible. Preventing new infections at the landscape level is the most effective way to manage *Heterobasidion* spp., though treatment decisions are usually made at the stand level (Zaluma et al. 2021). In relation to browsing damage, increased availability of palatable and preferred shrub and tree species (e.g. bilberry (*Vaccinium myrtillus*), rowan (*Sorbus aucuparia*), aspen (*Populus tremula*), willow (*Salix* spp.), and oak (*Quercus* spp.)), dispersed throughout the landscape rather than concentrated in one area, can result in decreasing overall browsing damage by large herbivores across the forest landscape (Bergqvist et al. 2018; Felton et al. 2022a). Conversely, scarcity of suitable resources leads herbivores to concentrate on stands with palatable species, resulting in intensified browsing pressure in those areas and their immediate surroundings (Kuijper et al. 2009; Bergqvist et al. 2018).

### Future disturbance trends

Climate change projections suggest future conditions will be more conducive to the life cycles of disturbance agents such as spruce bark beetle, along with worsening abiotic factors like drought in specific regions (Jaramillo et al. 2021). These changes are likely to amplify the effects of natural disturbances beyond historical norms (Seidl et al. 2008; Lindner et al. 2010). Global warming can also predispose trees to attack by pathogens, such as *Armillaria* spp., and susceptible forests will likely experience increased disease incidence and impacts (Sturrock et al. 2011). Furthermore, warmer winters may increase forest susceptibility to wind damage by shortening the soil frost period, potentially leading to a higher risk of tree uprooting during winter storms (Venäläinen et al. 2020). Overall, interactions among natural disturbances may intensify as the extent of susceptible and exposed forest areas increase (Ruel et al. 2023). In this context, proactive and effective adaptation strategies become of utmost importance in mitigating damage and maintaining forest productivity and resilience.

Climate change, in combination with increasing global trade, is expected to increase the likelihood of the introduction and establishment of new pests and pathogens (Liebhold et al. 2012; Santini et al. 2013; Szyniszewska et al. 2024). Due to the lack of co-evolution with native hosts, new pests and pathogens can cause large-scale mortality, and some have the potential to eliminate entire host species from affected regions (Waldron et al. 2008; Cleary et al. 2017). If a new pest or pathogen species becomes established, control measures aimed to reduce the impact may be implemented, and in some cases restoration may ultimately be required (Muzika 2017). Roberts et al. (2020) investigated how different silvicultural practices influence forest resilience to invasive pathogens, measured as primary and secondary infection rates and recovery. They found, for example, that secondary infection increased with tree density but decreased in mixed-species stands, while recovery improved after thinning and was also enhanced in mixed-species stands. However, responses in mixed-species systems were variable and depended on the particular tree species combination involved. Consistent with this, increased tree diversity has been observed to enhance invasibility at low levels of tree diversity, possibly through facilitation, while reducing invasibility at higher diversity levels, perhaps due to dilution effects (Guo et al. 2019). As for native pests and pathogens, it is expected that the effect of different forest adaptation strategies will ultimately depend on the biological characteristics of the particular species interacting with their hosts and their environmental context. More generally, reducing susceptibility to pests and pathogens may be supported by forest management strategies that promote vigorous trees and maintain a broad genetic base (Waring and O’Hara 2005).

In relation to browsing damage, the distributions of large herbivores in Europe are projected to shift northwards and to higher elevations in some locations in response to rising temperature and reduced snow cover (Felton et al. 2024c). Such shifts will likely alter damage patterns and, consequently, the suitability of adaptation strategies in some regions. In addition future forestry profit margins are expected to decline due to increasing and less predictable damage caused by natural disturbances, partly because the costs of unplanned harvesting and associated operations may rise if forests are not managed to enhance resistance (Skogsstyrelsen 2019). The future presents considerable uncertainty concerning natural disturbances—both those currently known and yet unknown disturbances. Therefore, it is advisable to develop and maintain resilient production forests capable of absorbing and recovering from a wide range of disturbances, thereby reducing impacts in the event of unforeseen events. At the same time, it must be acknowledged that such forest adaptations can have broader implications for other forest values, including biodiversity and ecosystem services, which should be taken into account in management decisions.

### Knowledge gaps

Most articles found in the systematic search covered wind, spruce bark beetle, root rot, and browsing damage, as major contributors to timber losses for forestry in Sweden. Conversely, fewer articles were found for snow damage, Scots pine blister rust, and European pine sawfly. Most likely, limited literature on these disturbances was found because of their lesser impact in terms of forest damage, for example, for the European pine sawfly or snow damage. The management of Scots pine blister rust lacks comprehensive guidelines despite recent observations of increased damage. However, recent advances found important genetic components that can be used for increasing the resistance of Scots pine (Persson et al. 2024). In addition, the search for *Armillaria* spp. also resulted in few articles likely due to significant research pre-dating the 2000s, conducted in other countries like Canada, and research focus on other tree species than Norway spruce and Scots pine. However, there is a larger research focus on *Heterobasidion* spp. root rot in Sweden due to large economic losses to the forest sector. Predominant adaptation strategies covered in the literature we found through the systematic search included mixed-species stands, short rotations, and few or no thinnings, while fewer articles covered longer rotations, logging residue removal, prescribed burning, and uneven-aged forestry. These topics constitute knowledge gaps that we recommend be addressed.

## Conclusions

The wide variety of disturbance agents and adaptation strategies makes it important to compile and review current knowledge in this field to support decision-making in forestry. The relationships found among forest characteristics, adaptation strategies and disturbance agents are complex, including variation in outcomes often occurring at different spatiotemporal scales (e.g., forest stand versus landscape level, seasonal versus annual dynamics). Our review indicates that certain forest adaptation strategies (e.g., mixed-species stands) have the potential to reduce forest susceptibility to multiple natural disturbances, suggesting promising opportunities for simultaneous risk mitigation. At the same time, implementing multiple management strategies at the landscape level is recommended, since no single approach is consistently beneficial or detrimental across all disturbance types. Our synthesis of the current literature suggests that combining several adaptation strategies over the rotation period might be desired if the stand in question is exposed to multiple disturbance agents. In addition, over the rotation period, the timing of the implementation of the strategies as well as the targeted disturbance needs to be considered to maximize the effectiveness of these strategies.

## Electronic supplementary material

Below is the link to the electronic supplementary material.

Supplementary file 1 (PDF 185 kb)

Supplementary file 2 (XLSX 29 kb)
