# Supplementary material for: Influence of management on susceptibility of Nordic boreal forests to damage by natural disturbances
Source: Ambio. 2026 May 19;55(11):2578–600. doi: 10.1007/s13280-026-02394-y (PMC13661854; doi:10.1007/s13280-026-02394-y)
Supplement: Supplementary file 1 [file 13280_2026_2394_MOESM1_ESM.pdf]

Article title: Influence of management on susceptibility of Nordic boreal forests to damage by natural disturbances

Journal name: Ambio

Author names: Teresa Fustel, Karin Öhman, Maartje Klapwijk, Tomas Lämås, Niklas Björklund, Johanna Boberg, Michelle Cleary, Annika Felton, Adam Felton, Stephanie Jonsson, Wiebke Neumann, Carin Nilsson, Åke Olson, Jonas Rönnberg, Jeannette Eggers

Affiliation and e-mail address of the corresponding author: Swedish University of Agricultural Sciences, teresa.fustel@slu.se

Supplementary material 1. Description of the search strings utilized to search for literature. The strings presented were developed to be used in WoS. Small adaptations not shown here were made to perform the same searches in SCOPUS database.

**String 1: Study population and geography**	"scots pine" OR "Norway spruce" OR "pinus sylvestris" OR "picea abies" OR "scotch pine" OR "P. sylvestris" OR "P. abies" OR Swed* OR Norw* OR Finland* OR Finnish* OR Scandinavia* OR Fennoscandia OR (*boreal* NEAR/5 (Swed* OR baltic* OR scandinav* OR fennoscand* OR "north* europ*" OR region OR forest)) OR *boreal* or temperate
**String 2: Natural disturbance**	▪ **Weather-related disturbances (wind and snow damage):** (wind* OR storm*) NEAR/5 (disturbance* OR impact* OR damag* OR "tree break*" OR uproot* OR "stem break*" OR bend* OR "forest damage*") OR "snow induce* damage*" OR (snow NEAR/5 (damage* OR load* OR heavy OR break* OR fall* OR cover* OR "tree break*" OR "heavy load*" OR "stem break*" OR bend* OR "forest damage*"))<br><br>▪ **Insect damage (spruce bark beetle, European pine sawfly, pine weevil):** "spruce bark beetle*" OR "ips typographus" OR "europe* sawfl*" OR "pine weevil*" OR herbivor* OR folivor* OR "neodriprion sertifer" OR "hylobius abietis"<br><br>▪ **Pathogen damage (root rot *Heterobasidion* spp., *Armillaria* spp., Scots pine blister rust):**<br>▪ heterobasidion<br>▪ armillaria<br>▪ cronartium<br><br>▪ **Browsing damage**: moose* OR alces OR elk OR cervus OR capreolus OR odocoileus OR deer* OR ungulat*) AND (damage OR "browsing damage*" OR browsing OR herbivore* OR "herbivore* damage*" OR brows*
**String 3: Adaptation strategy**	▪ **Mixed-species stands**: "mix*" OR "polyculture*" OR "admix*" OR "intermix*" OR "multi-species" OR "multiple species" OR "tree species diversity" OR "host diversity"<br><br>▪ **Rotation length (shorter and longer rotations)**: (Rotation* NEAR/5 (length* OR time* OR frequen* OR period* OR short* OR long* OR reduc* OR increase* OR extend*)) OR (harvest* NEAR/5 (length* OR time* OR frequen* OR period* OR short* OR long* OR rotation OR reduc* OR increase* OR extend*))

- **Few or no thinnings**: ((many OR several OR few OR number Or various OR numerous OR frequen* OR differen* OR change* OR increase* OR intensi* OR soft OR hard OR moderate OR reduce OR modif* OR high OR low OR no) NEAR/5 (“commercial thinning*” OR thinning* OR “intermediate harvest*” OR “tree removal*” OR “removal of tree*”)) OR “forest* thinning*” OR “commercial thinning*” OR “intermediate harvest*”

- **Logging residue removal**: (residu* NEAR/5 (harvest* OR extract* OR remov*)) OR (slash* NEAR/5 (harvest* OR extract* OR remov*)) OR (stump* NEAR/5 (harvest* OR extract* OR remov*)) OR (biofuel* NEAR/5 (harvest* OR extract* OR remov*)) OR (bio-fuel* NEAR/5 (harvest* OR extract* OR remov*)) OR (bio-energ* NEAR/5 (harvest* OR extract* OR remov*)) OR (bioenerg* NEAR/5 (harvest* OR extract* OR remov*)) OR (“whole-tree*” NEAR/5 (harvest* OR extract* OR remov*)) OR (“whole tree*” NEAR/5 (harvest* OR extract* OR remov*))

- **Prescribed burning**: “Burn* prescription” OR (prescrib* NEAR/5 (burn* OR fire*)) OR (“hazard reduc*” NEAR/5 (burn* OR fire*)) OR “control* burn*” OR swaling OR swailing OR “burn-off” OR “burn off” OR “back burn*” OR “back-burn” OR backfire

- **Uneven-aged forestry**: (forest* NEAR/5(“continu* cover*” OR “natural* regenerat*” OR multiage* OR alternativ* OR “common* sens*” OR unmanaged OR managed OR sustainabl*)) OR (silvicult* NEAR/5(“continu* cover*” OR “natural* regenerat*” OR multiage* OR alternativ* OR “common* sens*” OR unmanaged OR managed OR sustainabl*)) OR (regenerat* NEAR/5 (cut* OR fell* OR harvest* OR log*)) OR (select* NEAR/5 (cut* OR fell* OR harvest* OR log*)) OR (partial* NEAR/5 (cut* OR fell* OR harvest* OR log*)) OR (alternat* NEAR/5 (cut* OR fell* OR harvest* OR log*)) or (retent* NEAR/5 (cut* OR fell* OR harvest* OR log*)) OR (conserv* NEAR/5 (cut* OR fell* OR harvest* OR log*)) OR (gap* NEAR/5 (cut* OR fell* OR harvest* OR log*)) OR (patch* NEAR/5 (cut* OR fell* OR harvest* OR log*)) OR (dispers* NEAR/5 (cut* OR fell* OR harvest* OR log*))

Here follows a short example of how one of the 72 searches was performed. In this example, the search was prepared for WoS and covered the weather-related disturbances, wind and snow, and the mixed-species stands adaptation strategy.

“scots pine” OR “Norway spruce” OR “pinus sylvestris” OR “picea abies” OR “scotch pine” OR “P. sylvestris” OR “P. abies” OR Swed OR Norw* OR Finland* OR Finnish* OR Scandinavia* OR Fennoscandia OR (*boreal* NEAR/5 (Swed* OR baltic* OR scandinav* OR fennoscand* OR "north* europ*" OR region OR forest)) OR *boreal* or temperate* **(this is string 1 corresponding to study population and geography)**

AND **(this is the connector between string 1 and string 2)**

(wind OR storm*) NEAR/5 (disturbance* OR impact* OR damag* OR “tree break*” OR uproot* OR “stem break*” OR bend* OR “forest damage*”) OR “snow induce* damage*” OR (snow NEAR/5 (damage* OR load* OR heavy OR break* OR fall* OR cover* OR “tree break*” OR “heavy load*” OR “stem break*” OR bend* OR “forest damage*”))* **(this is string 2 corresponding to the natural disturbances wind and snow (weather-related))**

AND **(this is the connector between string 2 and string 3)**

“mix” OR “polyculture*” OR “admix*” OR “intermix*” OR “multi-species” OR “multiple species” OR “tree species diversity” OR “host diversity”* **(this is string 3 corresponding to the adaptation strategy of mixed-species stands)**
